# Contrasting Strategies of Photosynthetic Energy Utilization Drive Lifestyle Strategies in Ecologically Important Picoeukaryotes

**DOI:** 10.3390/metabo4020260

**Published:** 2014-04-29

**Authors:** Kimberly H. Halsey, Allen J. Milligan, Michael J. Behrenfeld

**Affiliations:** 1 Department of Microbiology, Oregon State University; 220 Nash Hall, Corvallis, OR 97330, USA; 2 Department of Botany and Plant Pathology, Oregon State University; 2082 Cordley hall, Corvallis, OR 97330, USA; E-Mails: allen.milligan@science.oregonstate.edu (A.J.M.); mjb@science.oregonstate.edu (M.J.B.)

**Keywords:** phytoplankton, metabolism, motility, *Micromonas pusilla*, *Ostreococcus tauri*, photosynthesis, energy utilization, carbon allocation, partitioning, primary production

## Abstract

The efficiency with which absorbed light is converted to net growth is a key property for estimating global carbon production. We previously showed that, despite considerable evolutionary distance, *Dunaliella tertiolecta* (Chlorophyceae) and *Thalassiosira weissflogii* (Bacillariophyceae) share a common strategy of photosynthetic energy utilization and nearly identical light energy conversion efficiencies. These findings suggested that a single model might be appropriate for describing relationships between measures of phytoplankton production. This conclusion was further evaluated for *Ostreococcus tauri* RCC1558 and *Micromonas pusilla* RCC299 (Chlorophyta, Prasinophyceae), two picoeukaryotes with contrasting geographic distributions and swimming abilities. Nutrient-dependent photosynthetic efficiencies in *O. tauri* were similar to the previously studied larger algae. Specifically, absorption-normalized gross oxygen and carbon production and net carbon production were independent of nutrient limited growth rate. In contrast, all measures of photosynthetic efficiency were strongly dependent on nutrient availability in *M. pusilla*. This marked difference was accompanied by a diminished relationship between Chl*_a_*:C and nutrient limited growth rate and a remarkably greater efficiency of gross-to-net energy conversion than the other organisms studied. These results suggest that the cost-benefit of decoupling pigment concentration from nutrient availability enables motile organisms to rapidly exploit more frequent encounters with micro-scale nutrient patches in open ocean environments.

## 1. Introduction

The flexibility of phytoplankton physiology enables their acclimation to environments that vary widely in resource concentrations. One well understood acclimation strategy is the adjustment of cellular pigment concentrations to available light [1,2,3] or nutrient [3,4,5,6,7] concentrations. This response is the reason why the ratio of chlorophyll_a to cellular carbon (Chl*_a_*:C) can, under certain conditions, be used as a diagnostic of nutrient limitation [3,8,9,10]. Other metabolic processes, including the rates of carbon fixation [11,12] and lipid biosynthesis [13,14,15] are also regulated in direct proportion to nutrient-driven growth rates. Understanding these and other acclimation strategies employed by phytoplankton is important for improving assessments of aquatic primary production and tools to manipulate algal growth for bioproduction purposes.

Considerable research effort has been dedicated to identifying cell properties expressed across taxonomic groups that can be directly related to abiotic factors. For example, the Chl-normalized ^14^C-uptake rate during short term incubations (< 12 h) increases with nitrate limited growth rate, despite Chl-normalized gross and net primary production being independent of growth rate. The sensitivity of ^14^C-uptake to growth rate is due to shifts in the proportion of newly fixed carbon allocation from short-lived end products in slow growing nitrate-limited cells to longer-lived polysaccharides in fast growing cells. These nutrient-dependent shifts in carbon utilization were directly linked to changing energy needs during different phases of the cell cycle [7]. Consequently, this effect can compromise interpretations of ^14^C-uptake data without a priori knowledge of a population’s growth rate [6].

The overall cellular reduction state also changes depending on nutrient status. This property is a measure of the relative proportion of energy rich to lower-energy oxidized carbon end-products [13]. Thus, in phytoplankton, the allocation of carbon and energetic currencies (ATP and NAD(P)H) to different metabolic pathways is tightly regulated to balance external resource availability with cell growth. Understanding the conditions leading to accumulation of lipids and other highly reduced compounds is of particular interest to bio-production efforts that use algal stocks [16]. Quantifying energy allocation is also vital to understanding phytoplankton responses to ongoing climate change [17,18,19] and is likely to play an important role in the productivity of biocenoses.

In previous work we were surprised by the striking similarity in patterns of photosynthetic energy allocation between two unrelated phytoplankton species, a green alga, *Dunaliella tertiolecta,* and a diatom, *Thalasiossira weissflogii,* across a wide range of steady state nitrogen limited growth rates [7]. Normalized to absorbed light, gross O_2_ production (GP_O2_^*^) was independent of growth rate in both species. GP_O2_^*^ is a measure of the total amount of energy available for the cell to use to carry out the complete set of metabolic processes leading to net growth (NP_C_^*^). Early in photosynthetic electron transport, a variety of different pathways can consume electrons prior to carbon fixation. Some of these pathways terminate with oxygen reduction, enhance the transmembrane proton gradient, and generate ATP (*i.e.*, Mehler reaction, midstream oxidase activity). Collectively, these pathways carry out light dependent respiration (LDR). NADPH formed through linear electron transport can also be used to reduce nitrate and sulfate. For both *D. tertiolecta* and *T. weissflogii*, when normalized to absorbed light, the energy allocated to major metabolic processes, including LDR, nitrate and sulfate reduction, and gross carbon production, did not change across growth rates or between species. Finally, a constant 30% of GP_O2_^*^ was retained as net carbon production in both *D. tertiolecta* and *T. weissflogii*, across all growth rates. These results suggested that a common energetic partitioning strategy may exist within phytoplankton. Importantly, underlying the growth rate-independent properties described above, are nutrient-dependent changes in elemental stoichiometry (C:N), pigment concentration, and allocation of carbon to pathways dedicated to ATP and/or NADPH production [7].

The study presented here extends our earlier work on photosynthetic energy allocation to *Micromonas pusilla* and *Ostreococcus tauri*, prasinophytes with differing geographic distributions and swimming abilities. The Prasinophyceae are picoeukaryotic phytoplankton (< 2 μm in diameter) that are widely distributed in marine ecosystems. In spite of their diminutive size, prasinophytes contribute a significant portion of marine carbon cycling [20,21]. Prasinophytes are structurally simple, containing a single chloroplast and mitochondrion, and exhibit substantial intraspecific morphological diversity [22]. *M. pusilla* RCC299 was isolated from the temperate ocean and has a single flagellum that is presumed to facilitate phototaxis [23,24] to optimize positioning for light in stratified water columns [25]. In contrast, *O. tauri* is nonflagellated and has a cosmopolitan presence in temperate waters. Species within the genera *Micromonas* and *Ostreococcus* diverge in traits that tune the cells to different light environments [26,27]. For example, Cardol *et al.* (2008) showed that *O. tauri* relies on relatively low pigment content and highly responsive nonphotochemical quenching to sustain growth at high light intensities (> 1500 μmol photons (m^2^ sec)^−1^). On the other hand, *Ostreococcus* RCC809 is a low light strain isolated from the base of the euphotic zone that maintains a high light absorption capacity [28] and appears to rely on midstream oxidase activity (e.g., PTOX) for ATP production and prevention of photoinhibition.

Here, we compare photosynthetic energy utilization in *M. pusilla* RCC299 and *O. tauri* RCC1558 grown over a wide range of steady state nutrient limited growth rates. We find that absorption-normalized gross oxygen and carbon production and net carbon production are independent of growth rate in *O. tauri*, a result that is nearly identical to our previous results for *D. tertiolecta* and *T. weissflogii*. In contrast, all measures of photosynthetic efficiency varied strongly with growth rate in *M. pusilla*. This marked difference was associated with nearly invariant pigment content across all nutrient limited growth rates in *M. pusilla*, and is a primary reason why photosynthetic efficiencies in this species decrease in parallel with growth rate. Furthermore, *M. pusilla* is ~ 20% more efficient at converting absorbed light to biomass. These results suggest that the cost-benefit of decoupling pigment concentration from nutrient availability enables motile organisms to exploit more frequent encounters with micro-scale nutrient patches in open ocean environments.

## 2. Results

**Figure 1:**
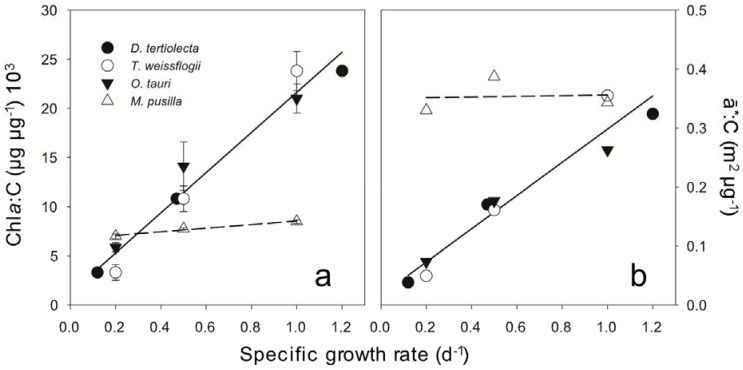
**(a)** The relationship between Chl*_a_*:C and nitrogen limited growth rate in *M. pusilla* (∆ and dashed line) contrasts with *O. tauri* (▼), *D. tertiolecta* (●), and *T. weissflogii* (○) given by the linear regression for all three species (solid line). **(b)** The relationships between carbon-normalized absorption and growth rate for all four species studied. Solid line is the linear regression for data for *D. tertiolecta* (●), and *T. weissflogii* (○) that were previously reported [6,7].

Cellular composition and photosynthetic electron utilization were evaluated in *M. pusilla* and *O. tauri* across steady state nitrate-limited growth rates ranging from 0.2 to 1.0 d^−1^. Carbon per cell decreased with increasing growth rate in both species (Table 1). Across all growth rates, cellular carbon in *O. tauri* was approximately half that of *M. pusilla*. Both prasinophytes had characteristically low ratios of Chl*_a_*:Chl*_b_* [29]; with this ratio nearing unity in *M. pusilla* (Table 1).The Chl*_a_* to carbon ratio increased with growth rate in *O. tauri*, reflecting both an increase in Chl*_a_* concentration and a decrease in C per cell with growth rate (Figure 1a). This result is essentially identical to our earlier findings for *D. tertiolecta* and *T. weissflogii* [6,7], with a combined relationship for all three species that is highly significant (r^2^ = 0.97) (Figure 1a). In stark contrast, there is not a clear correlation between Chl*_a_*:C and growth rate for *M. pusilla* (Figure 1a). In *M. pusilla*, C per cell decreased and cellular light absorption was invariant with growth rate (Table 1). Thus, under steady state growth conditions, this species does not down-regulate photosynthetic pigment concentration in proportion to nitrogen limited growth rate. Notably, Chl*_a_*:C for *M. pusilla* was similar to that for *O. tauri*, *D. tertiolecta*, and *T. weissflogii* at the slowest growth rates (Figure 1a), while carbon-specific absorption was similar for the four species at the highest growth rates (Figure 1b).

**Table 1:** Cell characteristics, light harvesting and fluorescence properties of steady state N-limited *M. pusilla* and *O. tauri* (values in parentheses are SE from triplicate cultures at each growth rate).

	*M. pusilla*			*O. tauri*		
Growth rate (d^−1^)	0.2	0.5	1.0	0.2	0.5	1.0
Cells ml^−1^ (× 10^7^)	1.6 (0.5)	2.1 (0.1)	2.2 (0.8)	2.3 (0.2)	4.7 (0.8)	3.1 (0.1)
C per cell (pg)	1.77 (0.45)	1.04 (0.20)	1.00 (0.41)	0.72 (0.03)	0.65 (0.19)	0.45 (0.03)
Cell volume (μm^3^)	2.9 (0.04)	3.6 (0.06)	4.5 (0.07)	nd^*^	nd	nd
Chl*_a_* per cell (fg)	11.9 (1.3)	10.2 (0.5)	8.5 (0.9)	4.2 (0.4)	7.4 (1.1)	8.1 (0.8)
C:N	7.27 (0.48)	5.22 (0.86)	3.56 (0.85)	11.0 (2.1)	7.40 (1.05)	4.38 (0.26)
N per cell (pg)	0.21 (0.02)	0.16 (0.01)	0.29 (0.04)	0.06 (0.02)	0.09 (0.01)	0.11 (0.07)
Chl*_a_*:Chl*_b_*	1.75 (0.04)	1.30 (0.12)	1.87 (0.04)	3.39 (0.22)	2.10 (0.36)	2.69 (0.15)
ā* (m^2^ mg Chl*_a_*^−1^)	0.048 (0.005)	0.050 (0.005)	0.037 (0.006)	0.013 (0.003)	0.028 (0.004)	0.021 (0.003)
F_v_/F_m_	0.33 (0.02)	0.38 (0.01)	0.48 (0.02)	0.52 (0.02)	0.52 (0.03)	0.56 (0.02)
F_v_/F_m_ + DCMU	0.35 (0.02)	0.41 (0.01)	0.50 (0.02)	0.52 (0.03)	0.52 (0.03)	0.56 (0.02)
σ_PSII_	900 (26)	1400 (110)	1300 (73)	1299 (60)	1084 (84)	1113 (92)

^*^not determined.

**Table 2:** Descriptions of production measurements and major metabolic pathways assessed in this study.

Abbreviation^1^	Production parameter	Units	Description	Method
F_v_/F_m_	Variable fluorescence	unitless	Efficiency with which light energy is trapped at PSII	FRRf (fast repetition rate fluorometry)
GP_O2_^*^	Gross O_2_ production	mmol O_2_ mmol photon^−1^	Total O_2_ produced at PSII	MIMS; ^16^O_2_ signal in the light + ^18^O_2_ signal in the light
LDR^*^	Light dependent respiration	mmol O_2_ mmol photon^−1^	O_2_ consumed by short water-water cycles, non-mitochondrial respiration	MIMS; ^18^O_2_ signal in light − ^18^O_2_ signal in the dark
DU_NS_	Direct utilization quotient		Fraction of GP_O2_ used directly for N and S reduction	Calculated from cellular N and GP_O2_
GP_C_^*^	Gross C production	mmol C mmol photon^−1^	Total energy available for carbon fixation	Calculated; see methods
	Net O_2_ production	mmol O_2_ mmol photon^−1^	Total O_2_ produced at PSII minus O_2_ consumed by respiration	MIMS; ^16^O_2_ signal in the light
NP_O2_^*^	Net O_2_ production (carbon-based)	mmol C mmol photon^−1^	Net O_2_ production minus energy used for N and S reduction	Calculated, see methods
	Mitochondrial respiration	mmol C mmol photon^−1^	Photosynthetic electron flow that passes through a carbon form and terminates with O_2_ reduction	Difference between GP_C_ and NP_O2_
	Catabolic reductant regeneration	mmol C mmol photon^−1^	Photosynthetic electron flow that passes through a carbon form and terminates with C reduction	Difference between NP_O2_ and NP_C_
NP_C_^*^	Net C production	mmol C mmol photon^−1^	Fixed carbon that is retained by the cell for the duration of the cell cycle	Growth rate cellular carbon

^1^ Throughout the manuscript, abbreviations with an asterisk (e.g., GP_O2_^*^) indicate the production parameter has been normalized to absorbed light.

Seven photosynthetic properties were evaluated at each growth rate and descriptions of these properties are summarized in Table 2. To compare photosynthetic properties between species and with our earlier results for *D. tertiolecta* and *T. weissflogii*, production values were normalized to light absorption. This normalization improves comparisons between species because it accounts for differences in pigment composition (Table 1) and thus reports on the efficiency of photosynthetic production per unit of light absorbed. In the following subsections, we focus on the similarities and differences in measured values of:

(1) Gross oxygen production (GP_O2_^*^)
(2) Light dependent respiration (LDR^*^)
(3) Gross carbon production (GP_C_^*^)
(4) Net oxygen production (NP_O2_^*^)
(5) Net carbon production (NP_C_^*^)

where the superscript asterisk denotes normalization to light absorption. Values reported are for oxygen or carbon production measured at the growth intensity (195–220 μmol photons m^−2^ sec^−1^). These data were used to assess relationships between photosynthetic properties and growth rate in each species and to compare patterns of productivity between species.

### 2.1. Production Efficiencies in O. tauri

**Figure 2:**
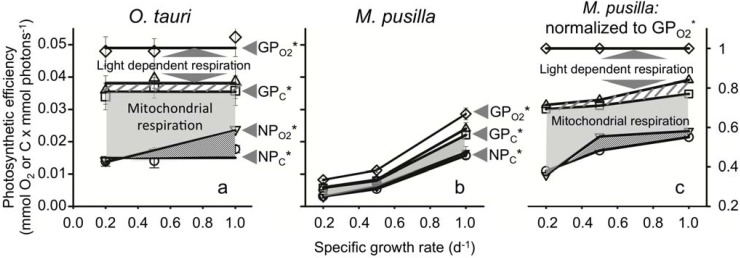
**(a)** Photosynthetic efficiencies across a wide range of nitrogen limited growth rates in *O. tauri* and **(b)** *M. pusilla*. Normalized to absorbed light, gross O_2_ production (GP_O2_^*^; ◇), light dependent respiration (GP_O2_^*^ − LDR^*^ (∆)), gross C production (GP_C_^*^; □), and net C production NP_C_^*^; ○) were independent of growth rate in *O. tauri*, but increase with growth rate in *M. pusilla.* Net O_2_ production (NP_O2_^*^; ▽) increased with growth rate in both strains and distinguishes the amount of transient carbon used for mitochondrial respiration (light grey shaded areas) from transient carbon catabolized for NADPH regeneration (dark grey area). Note that for *M. pusilla,* NP_O2_^*^ is only slightly greater than NP_C_^*^. **(c)** *M. pusilla* production values shown as their fractions of GP_O2_^*^ demonstrating a similar strategy of energy partitioning between species (compare a and c). The amount of energy dedicated to nitrate and sulfate reduction is indicated in a and c (striped area).

GP_O2_^*^ was invariant with growth rate and was 0.049 mmol O_2_ mmol photons^−1^ (Figure 2a). Photosynthetic energy used for light-dependent respiration was also independent of growth rate and accounted for 22% of GP_O2_^*^ (white region in Figure 2a). Using cellular nitrogen values, we estimated that photosynthetic energy used for nitrate and sulfate reduction accounted for 5% of GP_O2_^*^ (Figure 2a, striped region). The remaining 73% of GP_O2_^*^ is energy flow available for gross carbon production (GP_C_^*^; Figure 2a). As with GP_O2_^*^, LDR^*^, and GP_C_^*^, net carbon production (NP_C_^*^) was also independent of growth rate and was 0.015 mmol O_2_ mmol photons^−1^. This value is 30% of GP_O2_^*^ across all nitrogen limited growth rates (Figure 2a). The constancy of these values with growth rate means that *O. tauri* finely tunes its light harvesting capacity (Figure 1b) and photosynthetic production to precisely match demands for a given nitrate-limited growth rate while maintaining a relatively fixed partitioning between primary energy sinks. These results are highly consistent with findings for *D. tertiolecta* and *T. weissflogii.*

One photosynthetic property that did vary with growth rate was net O_2_ production. When this absorption-based production rate was converted to carbon equivalents (NP_O2_^*^), its value matched NP_C_^*^ at the lowest growth rate (Figure 2a). NP_O2_^*^ is higher than NP_C_^*^ at higher growth rates. The difference between NP_O2_^*^ and NP_C_^*^ represents a flow of electrons used for synthesizing more highly reduced organic molecules (e.g., lipids) [13,30].

### 2.2. Production Efficiencies in M. pusilla

In contrast to the majority of measurements described above for *O. tauri*, all photosynthetic properties increased with growth rate in *M. pusilla.* These behaviors were caused by cellular absorption that was independent of growth rate (Figure 1b). GP_O2_^*^ was only 0.008 mmol O_2_ mmol photons^−1^ in cells growing at 0.2 d^−1^. This value is ~6-fold lower than for *O. tauri.* In *M. pusilla*, GP_O2_^*^ increased to 0.029 mmol O_2_ mmol photons^−1^ in cells growing at 1.0 d^−1^, a value ~1.7-fold less than for *O. tauri* (Figure 2b). NP_C_^*^ increased from 0.003 at the slowest growth rate to 0.016 mmol C mmol photons^−1^ at the fastest growth rate, a value that matched NP_C_^*^ in *O. tauri* (Figure 2b). Thus, in *M. pusilla*, NP_C_^*^ was 38% to 55% of GP_O2_^*^ depending on growth rate. Interestingly, when each photosynthetic property is calculated as a fraction of GP_O2_^*^, the resulting energy utilization patterns are similar to those of *O. tauri* (Figure 2a,c).

### 2.3. Fluorescence Properties in Nutrient Limited O. tauri and M. pusilla

Differences in the regulation of light absorption in response to nutrient conditions were also observed in cellular fluorescence properties of *M. pusilla* and *O. tauri*. F_v_/F_m_ measures the efficiency with which light energy is captured at PSII, a property that most closely aligns with GP_O2_^*^. Therefore, it was not surprising that F_v_/F_m_ paralleled GP_O2_^*^ in both species. F_v_/F_m_ was maximal (~ 0.53) across all growth rates in *O. tauri* (Table 1). In contrast, F_v_/F_m_ decreased from 0.50 in fast growing *M. pusilla* to 0.38 and 0.35 in cells growing at the slower growth rates. We considered the possibility that this decrease in F_v_/F_m_ with growth rate was caused by incomplete reduction of the PQ pool during the FRRf protocol. Addition of 20 μM DCMU (an inhibitor of electron transfer from PSII to the plastoquinone pool) did not result in recovery of the fluorescence signal (Table 1). Thus, the growth rate dependent changes in F_v_/F_m_ in *M. pusilla* do not appear to reflect any limitation of the FRRf protocol in saturating PSII caused by incomplete reduction of the PQ pool.

Maximal F_v_/F_m_ values of about 0.50 and large antenna sizes (σ_PSII_) are characteristic for picoeukaryotes [31]. Values of F_v_/F_m_ for larger algae (diatoms and chlorophytes) growing under steady state conditions are typically 0.55 to 0.65 (reviewed in [32]). Lower F_v_/F_m_ values in very small cells (excepting the cyanobacterium *Prochlorococcus marinus* [33] have been discussed before for cyanobacteria [32] and are most likely a result of contaminating minimum yield fluorescence (F_O_) from PSI [34]. In changing nutrient conditions, there are energetic benefits (lower costs) associated with increasing σ_PSII_ instead of numbers of reaction centers, especially for picoeukaryotes that tend to dominate in nutrient limiting environments [35]. These considerations suggest that there was evolutionary pressure for large σ_PSII_ in picoeukaryotes, but despite shared phylogeny, there are clearly different light harvesting strategies for the two prasinophytes studied here during nitrogen-limited growth.

### 2.4. Photosynthetic Energy Allocation

**Figure 3:**
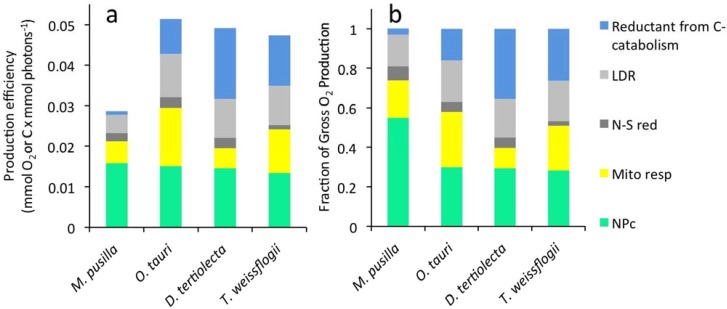
(**a**) Photosynthetic electron allocation to major metabolic sinks whose sum is GP_O2_^*^. For all four algal species, data are shown for cells growing at 1.0 d^−1^. Photosynthetic energy allocated to each of five metabolic sinks. (**b**) Fraction of gross photosynthesis (GP_O2_) allocated to the five metabolic sinks. Note that data shown for *D. tertiolecta* and *T. weissflogii* were collected in previous studies [6,7].

Differences in photosynthetic energy allocation to major metabolic sinks were also observed between the four phytoplankton species studied. GP_O2_^*^ is the sum of LDR^*^, mitochondrial respiration, catabolically regenerated reductant, and NP_C_^*^. We have now completed this suite of measurements for *M. pusilla*, *O. tauri, D. tertiolecta,* and *T. weissflogii.* Energy allocation to these major metabolic sinks is shown in Figure 3 for cells grown at the fastest growth rates for all four species. We find that NP_C_^*^ is nearly identical for all four species (green bar in Figure 3a). However, GP_O2_^*^ for *M. pusilla* is roughly half that for the other three species (compare total bar heights in Figure 3a). This result implies that, relative to the other species, *M. pusilla* has evolved to minimize energy allocation to pathways other than NP_C_^*^ during rapid growth. In other words, *M. pusilla* has a high gross-to-net photosynthetic conversion efficiency (Figure 3b).

The difference between GP_O2_^*^ and NP_C_^*^ corresponds to photosynthetic energy that is lost during the timeframe of a cell cycle. Some of this energy is consumed by light-dependent respiration and N and S reduction (dark and light grey bars in Figure 3). The relative fraction of electrons dedicated to these pathways is similar for the four species studied (Figure 3b). Carbon fixed by the Calvin cycle that is not retained as NP_C_^*^ is catabolized to CO_2_ either by mitochondrial respiration or the oxidative pentose phosphate pathway. Transient carbon flow through these pathways is represented in Figure 3 by the yellow and blue bars. Both of these pathways yield ATP, but the latter also produces NADPH (reductant) that is needed for synthesis of carbon end-products, such as lipids and nucleic acids, that are more biochemically reduced than glyceraldehyde 3-phosphate, the initial product of the Calvin Benson cycle. Since this process does not consume oxygen, electron flow allocated to reduction of existing carbon forms is registered in measurements of NP_O2_^*^. In combination with the other measurements made in this study, net O_2_ production can be used to quantify the amount of energy allocated to mitochondrial respiration and to catabolism for reductant regeneration.

The difference between GP_C_^*^ and NP_O2_^*^ is photosynthetic energy dedicated to mitochondrial respiration (grey shaded area in Figure 2). Energy flow to mitochondrial respiration decreased with increasing growth rate in both *O. tauri* and *M. pusilla*. This trend was also found for *D. tertiolecta* and *T. weissflogii* [7]. However, the relative magnitude of this metabolic sink differed significantly between species (Figure 3b).

The difference between NP_O2_^*^ and NP_C_^*^ is the amount of energy dedicated to reductant regeneration by carbon catabolism (dark grey area in Figure 2). In this case, energy allocated to reductant regeneration increased with growth rate in all species (Figure 2, [7]). However, energy allocation to this metabolic sink was dramatically diminished in *M. pusilla* relative to *O. tauri* and the other species (blue bars in Figure 3). In cells growing at 1.0 d^−1^, only 3% of GP_O2_^*^ was dedicated to regenerating reductant in *M. pusilla* compared to 16% in *O. tauri* (Figure 3b).

## 3. Discussion

Understanding and comparing photosynthetic energy utilization strategies in different phytoplankton species is important for interpreting field and laboratory production measurements, model and satellite assessments of global ocean photosynthesis, and for bioenergy applications. Our previous work with a diatom, *T. weissflogii*, and a green alga, *D. tertiolecta*, put forth the possibility that a single model may be sufficient to describe fundamental principles of algal energy allocation during steady state nutrient limited growth [7]. In this initial model, concentrations and activities of light harvesting, photosynthetic electron transport, and carbon metabolic components are tuned such that key measures of photosynthetic efficiency (GP_O2_^*^, LDR^*^, GP_C_^*^, and NP_C_^*^) do not vary with nutrient-limited growth rate (*i.e.*, see Figure 2a). Furthermore, the relationships between these parameters suggested that a shared strategy of energy economy dictates energy expenditures. This strategy involves investment of 25% of GP_O2_^*^ into non-carbon fixing pathways (LDR and N and S reduction) and 45% into “transient” carbon products (carbon forms that are oxidized to CO_2_ via mitochondrial respiration or the oxidative pentose phosphate pathway within the timeframe of the cell cycle). Finally, net carbon production accounts for 30% of GP_O2_^*^. The quantitative similarities in photosynthetic energy allocation between the two species, *D. tertiolecta* and *T. weissflogii,* provided an approach for system-level modeling of photosynthate utilization in phytoplankton that is based on energetic stoichiometry.

### 3.1. Contrasting Strategies of Photosynthetic Energy Allocation

Here, we show that this strategy of photosynthetic energy utilization is also used by *O. tauri*. Both the growth rate-independent behavior of key photosynthesis properties and general energy allocation to major metabolic sinks were consistent with results for *D. tertiolecta* and *T. weissflogii. O. tauri* also exhibited a consistent gross-to-net ratio of 3.3. This result provides further support for the idea that a standard value approximating 3.3 can be used to estimate NP_C_^*^ based on GP_O2_^*^ determinations in the field or via satellite retrievals (GP_O2_^*^:NP_C_^*^ = 3.3) [36], at least in areas where nitrogen availability limits growth rate. Furthermore, these results suggest that the efficiency with which harvested energy is converted to biomass is a conserved relationship and independent of taxonomic delineations. This idea is not only important for improving ecosystem models of primary production, but also for estimates of biomass production from algal stocks for biofuels and other economically desirable products. Such conversion factors provide key constraints to cell-level models for designing optimized systems of algal bio-production [37,38].

However, our new results for *M. pusilla* for which GP_O2_^*^: NP_C_^*^ varied from 1.8 to 2.7 depending on growth rate, suggest that the use of conversion factors may require knowledge of the phytoplankton community composition. A similarly low gross-to-net ratio (high energy conversion efficiency) was exhibited by the diatom, *Phaeodactylum tricornutum*, grown under dynamic light conditions [39]. For both *M. pusilla* and *P. tricornutum,* the low gross-to-net ratios are primarily attributed to an extremely diminished requirement for catabolically regenerated reductant (blue bar in Figure 3).

### 3.2. Cellular Requirements for NADPH

Photosynthetic electron flow that is fixed into a carbon form and then later catabolized, probably via the oxidative pentose phosphate pathway, regenerates reductant (NADPH) for biosynthesis of more reduced carbon forms (e.g., lipids, nucleic acids) [6,13]. Thus, species with high lipid content are expected to show greater disparity between NP_O2_^*^ and NP_C_^*^, and lipid content for some species varies depending on growth condition [16,40]. Under steady state nitrogen limited growth, phospholipids were 65%–75% of the cellular lipid pool in *D. tertiolecta* [13]. Considering that phospholipids are membrane lipids external to the chloroplast, and that *M. pusilla* and *O. tauri* are the smallest known free-living eukaryotic cells with simple cellular structures (only a single mitochondria), it may not be surprising that reductant regeneration is a smaller fraction (3% and 16% in these two species, respectively, growing at 1.0 d^−1^) of GP_O2*_ than in the larger algae (blue color in Figure 3b). For *M. pusilla*, the diminished requirement for regenerated reductant across all growth rates underlies a significantly greater gross-to-net energy conversion efficiency.

Other metabolites, such as osmolytes, may serve to influence the requirement for reductant regeneration. It is tempting to hypothesize that osmolytes serve as a temporary energy storage pool. Phytoplankton produce osmolytes to maintain intracellular osmotic concentrations that are slightly greater than the surrounding seawater. Osmolytes are low molecular weight and highly reduced carbon compounds that can reach intracellular concentrations of 100–530 mM in phytoplankton [41,42]. Even higher concentrations have been discussed under nutrient limited growth [42,43]. Trimethylamine oxide, glycine betaine, proline, DMSP, and many other zwitterionic compounds can function as osmolytes [41], but DMSP is the most well-studied for its potential role in climate regulation. These compounds may serve additional roles in protection against grazing [44] and as an energy overflow mechanism during periods of low nitrogen availability and saturating light intensities [42,45]. Algal species use different strategies to regulate osmolyte concentrations [41], but their physiological roles have certainly not been fully elucidated. We suggest that these molecules may provide accessible short-term (transient) energy storage. Cell growth models based on optimal resource allocation commonly include a carbon storage pool [46,47]. Adding an energy storage pool may improve agreement between observed and predicted behaviors. It may be useful in future studies on photosynthetic electron utilization to assess intracellular osmolyte concentrations and their turnover rates to better understand their growth rate-dependent regulation.

Nucleic acid biosynthesis is another, more obvious, reductant requiring pathway. Consistent with the growth rate dependent increase in magnitude of the “regenerated reductant” metabolic sink, cultures of rapidly dividing cells have a greater amount of nucleic acid than slow growing cells. Accordingly, the overall magnitude of the “regenerated reductant” metabolic sink may be affected by genome size. For the four species studied, the magnitude of this sink decreases in the following order *D. tertiolecta* > *T. weissfloggi* > *O. tauri* > *M. pusilla* (Figure 3); but genome sizes are *T. weissfloggi,* est. 680 Mb > *D. tertiolecta*, est. 300 Mb > *M. pusilla,* 20.9 Mb [27] > *O. tauri,* 12.5 Mb [48]. Therefore, genome size does not appear to be a predominant factor in determining the amount of energy required for reductant regeneration.

Interestingly, for all four species the growth rate dependent increase in energy allocated to reductant regeneration is coupled to a corresponding growth rate dependent decrease in mitochondrial respiration (Figure 2, [7]). The energetic product of mitochondrial respiration is ATP, and the oxidative pentose phosphate pathway produces ATP and NADPH in proportions dictated by cell requirements for biosynthesis. In slow growing nitrogen limited cells, energetic requirements are dominated by maintenance activities, which are primarily fueled by ATP. Cells obtain ATP for maintenance activities from both carbon and non-carbon (LDR) pathways. However, energy allocated to LDR^*^ was essentially the same for all species studied and across all growth rates. Despite differences between the four species in the amount of carbon allocated to mitochondrial respiration and reductant regenerating pathways, the total amount of transient carbon remains nearly the same across all growth rates. Therefore, it appears that the cellular balance of ATP and NADPH is controlled at the level of carbon metabolism.

### 3.3. Light Harvesting: Tuning vs. Priming

Differences in the light harvesting properties for *O. tauri* and *M. pusilla* are primary factors influencing the contrasting photosynthetic efficiencies of the two species. In *O. tauri,* ā*:C decreased with decreasing growth rate. Thus, similar to other species growing under steady-state nitrogen limitation [6,49], *O. tauri* tunes its light harvesting capacity in direct proportion to the nutrient environment, causing F_v_/F_m_ to be maximal and GPP_O2_^*^ and NP_C_^*^ to be constant across all growth rates. In contrast, ā*:C remained constant with growth rate in *M. pusilla*, but F_v_/F_m_ and σ_PSII_ declined. One explanation for these data is that *M. pusilla* maintains pigment complexes that are detached from the reaction centers at low growth rates. Detached pigment complexes do not yield variable fluorescence, but may contribute to F_O_, and can thus, cause depressed F_v_/F_m_ values. Phytoplankton have been shown to harbor detached pigment complexes during iron limited, nitrogen replete growth conditions [50]. This strategy may allow cells to respond quickly to pulses of iron deposition [18]. It is possible that *M. pusilla* uses a similar strategy during nitrogen limited growth. Namely, by harboring “reserve” light harvesting capacity, cells in nitrogen depleted waters are primed to rapidly respond to sporatic pulses of nitrogen (see below).

Maintenance of ‘excess’ light harvesting capacity in very slow growing *M. pusilla* almost certainly requires an active light energy dissipation system to protect cells from oxidative stress (note, only a small fraction of the energy absorbed by these complexes is re-radiated as fluorescence). Absorbed light that is not used for photosynthesis, or lost as fluorescence, can be thermally dissipated through non-photochemical quenching (NPQ) mechanisms. Activity of NPQ is typically associated with exposure to high light intensities, but in our experiments, cultures were grown under constant illumination at ~ 200 μmol photon m^−2^ s^−1^, an irradiance that is not growth-inhibitory [51]. Nevertheless, the decline in gross O_2_ production per unit absorption (GPP_O2_^*^) implies that as nutrient limited growth rate decreases, an increasing amount of absorbed light is likely dissipated via the xanthophyll cycle. Xanthophylls are carotenoid pigments that alternate between epoxidation and de-epoxidation states to dissipate excess light energy. The pigment complexes maintained by iron-limited phytoplankton are also associated with strong NPQ responses [50].

The ā*:C was similar for all four species studied at the fastest growth rate. If we assume that *M. pusilla* growing at the fastest growth rate has optimized ā*:C such that NPQ is minimized, then the ratio of ā*:C for *M. pusilla* to ā*:C for *O. tauri* at each specific growth rate (x) yields

*y* = 0.98*x*^−12^ (1)

where *y* is pigment in excess of that needed for photochemistry and increases exponentially from the minimum value of 1 at a specific growth rate of 1 d^−1^. Thus, *y* is also proportional to the amount of absorbed light energy that is dissipated by NPQ. *M. pusilla* maintains relatively high photoprotective pigment to Chl*_a_* ratios irrespective of light intensity [52]. Although the details regarding pigment regulation in *M. pusilla* are not known, these results suggest that this species has dispensed with processes needed to adjust cellular content of light harvesting and photoprotective carotenoid pigments in response to fluctuating light and nutrient conditions.

### 3.4. Linking Motility to a New Photosynthetic Energy Utilization Strategy

It appears that in nitrogen-depleted waters, *M. pusilla* harbors excess light harvesting capacity to rapidly respond to fluctuations in nitrogen availability. Some flagellates may have evolved this strategy to exploit micro-scale nutrient patches. Studies evaluating the cost-benefits of motility have mainly focused on motility as a strategy to optimize light exposure within the vertical water column [25]. The ability to vertically migrate over the diel cycle allows cells to avoid periods of excessive light that lead to photoinhibition as well as to access deeper waters where nutrient resources are present at higher concentrations than at the surface. Raven and Richardson (1984) concluded that the costs associated with flagellar synthesis, assembly and operation outweigh the benefits associated with increased nutrient access, especially in small cells [53]. However, another study suggested that the estimated cost for *M. pusilla* migrating at about 100 μm sec^−1^ [24] is only 1%–3% of mitochondrial respiration rates [23].

Motile *M. pusilla* and nonmotile *O. tauri* can experience starkly different nitrogen concentrations during the duration of a cell cycle. The single-cell view of the marine environment is spatially and temporally heterogenous in nutrient concentration [54]. Recent modeling studies have demonstrated that motile microbes use chemotaxis to target micro-scale patches of high nutrient concentration in marine environments while nonmotile microbes are dependent on diffusional forces to drive their nutrient experiences [55]. In the ocean, the spatial scales of relevant nutrient gradients are in the range of 10–300 μm, well within the range of motile flagellates. Several important time-scales determine nutrient patch accessibility to motile microbes: (1) swimming speed; the time required for an organism to reach the highest concentration of a nutrient patch, (2) the lifetime of the nutrient patch, and (3) the rate of nutrient patch genesis. The swimming speed for *M. pusilla* is approximately 100 μm sec^−1^ which is similar to speeds of 70–90 μm sec^−1^ measured in toxic *Heterosigma akashiwo* [56]. Dinoflagellates average slightly faster swimming speeds of ~230 μm sec^−1^ [57]. Nutrient patches persist for up to 60 sec [55].

These time-scales suggest that removing nutrient-dependent regulation of pigment synthesis and, instead, maintaining a high light harvesting capacity, allows *M. pusilla* to immediately realize the benefits of encountering a high nutrient patch. The high efficiency of gross-to-net energy conversion exhibited by *M. pusilla* creates a direct energetic benefit to the associated trade-off. These results suggest that the productivity of *M. pusilla* depends on the frequency of encounters with and duration of exposure to nutrient patches. As *M. pusilla* migrates between nutrient patches, NP_C_^*^ fluctuates such that it is highest during encounters with high nutrient concentrations. On the other hand, *O. tauri* up- or down-regulates its light harvesting capacity to a relatively constant and low nutrient concentration environment that is controlled by diffusive properties, and its NP_C_^*^ remains constant. For *M. pusilla,* the costs associated with maintaining a high light harvesting capacity appear to be outweighed by the potential energetic benefit associated with motility.

## 3. Experimental Section

*M. pusilla* RCC299 was kindly provided by A. Worden (Monterey Bay Aquarium Research Institute). *M. pusilla* RCC299 and *O. tauri* RCC1558 were grown at 20°C in nitrate-limited steady state chemostats. Chemostat culturing provides a powerful system for studying phenotypic plasticity [58] under nutrient conditions that are reasonably reflective of open ocean environments. Cultures were grown in 24 h constant cool white fluorescent light at near saturating irradiance of 190–215 μmol photons (m^2^ sec)^−1^ as measured with a quantum meter and 4π spherical quantum sensor (Biospherical Instruments, model QSL-100, San Diego, CA, USA). Growth medium for both species was artificial seawater K medium with 882 μM NaNO_3_ added as the limiting nutrient and NaH_2_PO_4_ increased to 155 μM. The growth medium was supplied from a reservoir to cultures (0.3 L) at flow rates calculated to result in specific growth rates of 0.2, 0.5 and 1.0 d^−1^ according to the equation:

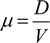
(2)
where μ is specific growth rate (d^−1^), D is flow rate (L d^−1^), and V is culture volume (L) maintained by siphon tubing. Cultures were aerated by bubbling with filtered air. Following at least seven generations, cell density and chlorophyll concentration were measured daily. When these values were stable (variation < 5%) for 3 consecutive days, cultures were considered to be in steady state, and measures of production and other physiological properties were conducted.

For *M. pusilla* RCC299, triplicate measurements of cell density, diameter, and volume were made using a Multisizer 3 Coulter counter equipped with a 25 μm aperture (Beckman Coulter; Miami, FL). Measurements of *O. tauri* RCC1558 culture density were made using a FACScan flow cytometer (Becton Dickinson; Franklin Lakes, NJ). Chlorophyll concentrations were determined from 3 mL samples filtered onto 25 mm glass fiber filters (Whatman GF/F) that were extracted overnight in 90% acetone at -20°C. Filters used for *O. tauri* RCC1558 were precombusted to decrease the nominal pore size to ~0.45 μm and prevent cell loss. Chlorophyll a and b were quantified using absorption values measured at 647 and 664 nm with absorption at 750 nm used as the blank [59]. Spectrally averaged cross sectional area (ā^*^; used to calculate absorbed light, [60]) was measured using the filter pad method with appropriate path-length corrections [61]. The spectral distribution of light was measured with a spectroradiometer (Ocean Optics USB2000; Dunedin, FL, USA). All production measurements were normalized to absorbed light according to the equation

P* = *P*(*Chl_a_* ∙ *I_g_* ∙ ā*)^−1^ (3)

where P^*^ is photosynthetic efficiency in units of mmol O_2_ or C (mmol photons)^−1^, P is the rate of oxygen or carbon production, Chl*_a_* is the concentration of chlorophyll a, I_g_ is the growth irradiance, ā^*^ is the spectrally averaged cross sectional area per unit Chl*_a_*.

For CHN analysis, 2, 3, and 4 mL samples were filtered onto precombusted 25 mm GF/F filters to ensure a linear relationship between C or N and volume filtered. Filtrate (4 mL) was also filtered and the recovered C or N values subtracted from sample mass values. Cellular C and N were measured with an Exeter Analytical EA1 elemental analyzer (North Chelmsford, MA). By definition, net carbon production (NP_C_) is the product of μ and cellular carbon. As described previously [6,7], the fraction of gross photosynthetic electron flow used directly for N and S reduction (*DU_NS_*) was calculated using cellular N and an N:S ratio of 16:1.3 [62] according to the equation

*DU_NS_* = [(2 ∙ *μ* ∙ (*N* + *S*)) + *GP*_*o*2_] ∙ (*GP*_*o*2_)^−1^ (4)

where measurement of GP_O2_ is described below. These *DU_NS_* values were used to calculate GP_C_ according to the equation

*GP_c_* = (*GP*_*o*2_ − *LDR*) ∙ (*DU_NS_*)^−1^ (5)

where light dependent respiration is LDR and measured as described below.

Membrane inlet mass spectrometry (MIMS) was used to measure rates of oxygen production and consumption in steady state cultures of *M. pusilla* RCC299 and *O. tauri* RCC1558. This system consists of a 5 mL water-jacketed incubation chamber that was maintained at 20°C and continuously mixed using a magnetic stir bar. Cell suspensions were exposed for 3–5 min to 7 light levels (0–750 μmol photons (m^2^ sec)^−1^) alternating with 1–2 min dark periods. Measurements were concluded with a 4–6 min dark period. To distinguish mitochondrial respiration and light dependent respiration, ^18^O_2_ was added to the chamber as a tracer (the rate of ^18^O_2_ consumption in the light is slightly greater than in the dark). A teflon membrane stretched over a tube inserted into the chamber allows oxygen and argon to be detected by a Prisma QMS-200 (Pfeiffer) quadrapole mass spectrometer with closed ion source and electron multiplier detector recording at mass/charge ratios of 32 (^16^O_2_), 36 (^18^O_2_), and 40 (Ar). Oxygen signals were calibrated with O_2_–saturated K-media and zero-O_2_ K-media (+ sodium dithionite) and normalized to Ar. We verified that cellular ^18^O_2_ consumption matched ^16^O_2_ consumption in the dark when the initial fraction of ^18^O_2_ was varied from 38–90 μM. To account for changes in isotope dilution throughout the experiment, rates of oxygen production and consumption were calculated by dividing the observed rates by the fraction of ^16^O_2_ or ^18^O_2_ present during exposure to each light level. Photosynthesis-irradiance (PE) relationships for gross and net O_2_ production were fit to measured data using non-linear least squares regression analysis and a hyperbolic tangent model [63] yielding O_2_-production rates for the growth irradiance. Therefore, production values do not reflect maximum photosynthetic efficiencies, which would be calculated from the light-limited slope of photosynthesis-irradiance curves Net O_2_ production rates were converted to C units using *DU_NS_* values to allow comparison with gross and net carbon production. Accordingly, C-based net O_2_ production (NP_O2_) was calculated as

*NP*_*o*2_ = (net oxygen production) ∙ *DU_NS_*^−1^ (6)

Variable fluorescence (F_v_/F_m_ = (F_m_−F_o_)/F_m_) and the effective absorption cross section of PSII (σ_PSII_) were measured using a fast-repetition-rate fluorometer (FRRf; [64]). Samples were dark acclimated for 5 min prior to measurement of F_o_ (Chl fluorescence yield when all functional PSII reaction centers are oxidized) and F_m_ (Chl fluorescence yield when all functional PSII reaction centers are reduced). We verified that measurements did not change when the dark acclimation period was extended to 10, 15, and 20 min.

## 4. Conclusions

This study demonstrated that very different strategies of photosynthetic energy utilization operate in taxonomically related phytoplankton that are abundant members of open ocean natural communities [65]. These contrasting growth strategies were elucidated using multiple measures of photosynthetic activity. Assessing physiological traits and their associated trade-offs is an important goal in understanding phytoplankton ecophysiology [66]. We suggest that despite occupying the same environmental niche [65], prasinophytes partition available environmental resources by using very different photosynthetic energy allocation strategies. These allocation patterns are governed by metabolic mechanisms that can be shared between unrelated algal species [7] or uniquely associated with specific physiological traits, such as motility, as suggested by the results for *M. pusilla* in this study.
